# Toxicosis by Plant Alkaloids in Humans and Animals in Colombia

**DOI:** 10.3390/toxins7124892

**Published:** 2015-12-11

**Authors:** Gonzalo J. Diaz

**Affiliations:** Laboratorio de Toxicología, Facultad de Medicina Veterinaria y de Zootecnia, Universidad Nacional de Colombia, Bogotá, Colombia; gjdiazg@unal.edu.co; Tel.: +57-1-316-5000 (ext. 19450)

**Keywords:** toxic alkaloids, toxic plants, Colombia

## Abstract

Due to its tropical location, chains of mountains, inter-Andean valleys, Amazon basin area, eastern plains and shores on both the Atlantic and Pacific Oceans, Colombia has many ecosystems and the second largest plant biodiversity in the world. Many plant species, both native and naturalized, are currently recognized as toxic for both animals and humans, and some of them are known to cause their toxic effects due to their alkaloid content. Among these, there are plants containing the hepatotoxic pyrrolizidine alkaloids, neurotoxins such as the indolizidine alkaloid swainsonine and the piperidine alkaloids coniine and γ-coniceine and tropane alkaloids. Unfortunately, the research in toxic plants in Colombia is not nearly proportional to its plant biodiversity and the scientific information available is only very scarce. The present review aims at summarizing the scarce information about plant alkaloid toxicosis in animals and humans in Colombia.

## 1. Introduction

Colombia has the second largest plant biodiversity in the world. From approximately 300,000 vascular plant species reported worldwide, 25,000 (8%) have been reported in Colombia [1]. Toxicosis by plants in animals and humans in Colombia is, therefore, not uncommon. In domestic animals, toxicosis can result from different circumstances of exposure. For example, pets can have access to toxic ornamental plants at home, whereas herbivores (mainly cattle and horses) may be exposed by consuming toxic plants present in grazing lands. Humans are exposed to toxic plants either by voluntary intake when plants are used as some kind of folk remedy, or accidentally when toxic plants are mistaken for edible plants. Another type of exposure is the intentional administration of tropane alkaloids from *Brugmansia* spp. for criminal purposes.

Unfortunately, many of the toxicoses associated with plants in Colombia are not documented in the scientific literature. Only recently the first book on toxic plants of Colombia was published [2]. In this book, 150 plant species corresponding to about 100 genera and 34 botanical families are mentioned as potentially toxic and some of them are toxic due to their content of alkaloids (mainly pyrrolizidine, tropane and piperidine alkaloids). The aim of the present review is to summarize the scarce information about toxicosis by plant alkaloids in Colombia in both animals and humans. Intoxication by so-called “recreational drugs” or by “drugs of abuse” is not discussed in the present review. Table 1 summarizes the information so far available in Colombia.

**Table 1:** Major alkaloid containing plants affecting animals and humans in Colombia.

Botanical Family	Binomial and Main Common Name	Major Effects	References
Apiaceae	*Conium maculatum* (cicuta)	Contains piperidine alkaloids (mainly coniine and γ-coniceine). Has been associated with accidental and deliberate deaths in humans (postmortem finding).	[1,3]
Asteraceae	*Senecio formosus* (árnica)	Contains pyrrolizidine alkaloids, particularly lycopsamine. Affects humans when ingested as an infusion. Produces veno-occlusive disease. Safe when used topically.	[2,3,4,5,6]
	*Senecio madagascariensis*, *S. vulgaris* (manzanilla)	Contain pyrrolizidine alkaloids. Cause liver damage in herbivores. Represent a serious problem as weeds in pastures.	
Boraginaceae	*Symphytum officinale* (consuelda)	Contains pyrrolizidine alkaloids, especially retrorsine. Affects humans when ingested as an infusion. Produces veno-occlusive disease. Safe when used topically.	[1,3]
	*Heliotropium europaeum*, *H. indicum* (rabo de alacrán)	Contain pyrrolizidine alkaloids. Affect grazing herbivores when present as a weed in pastures.	[7,8,9]
Convolvulaceae	*Ipomoea carnea* (batatilla)	Contains an indolizidine alkaloid known as swainsonine. Affects mainly cattle from the eastern plains when present in pastures.	[10]
Fabaceae	*Crotalaria* spp. (cascabelito)	Contain pyrrolizidine alkaloids, especially monocrotaline and usaramine. The seeds can contaminate agricultural crops and animal feeds. Crotalarias have caused severe outbreaks of toxicosis in pigs and poultry in Colombia.	[11,12,13,14]
Solanaceae	*Brugmansia* spp. (borrachero)	Contain tropane alkaloids (scopolamine and atropine). Affect humans. Extracts of these plants are used for criminal purposes.	[15]
	*Datura* spp. (chamico)	Contain tropane alkaloids (scopolamine and atropine). The seeds can contaminate agricultural crops and animal feeds.	

## 2. Pyrrolizidine Alkaloids

Pyrrolizidine alkaloids (PAs) are a large group of hepatotoxic, pneumotoxic and genotoxic compounds with a pyrrolizidine group in their structure [16]. The chemistry and toxicology of PAs has been extensively reviewed elsewhere [16,17,18]. The 1,2-dehydropyrrolizidine ester alkaloids (DHPAs) and their *N*-oxides occur in about 3% of the flowering plants and all are considered hazardous if ingested [16,17]. DHPAs are converted to pyrrolizine esters by cytochrome P450 enzymes and these compounds, also known as “pyrrolic” esters, are unstable electrophilic alkylating molecules [16]. In general, PAs induce hepatocyte necrosis that progresses to liver failure. Some PAs are also potent carcinogens at levels below those causing hepatic necrosis while others can affect the lungs (especially monocrotaline) [17,18].

The main botanical families with plants containing PAs are Asteraceae (e.g., *Senecio* spp.), Boraginaceae (e.g., *Heliotropium* spp., *Symphytum* spp.) and Fabaceae (e.g., *Crotalaria* spp.) [18]. Plants belonging to these three families can be found in Colombia as either native or naturalized species and some have been associated with toxicosis in animals and humans.

### 2.1. Pyrrolizidine Alkaloid Toxicosis in Humans

*Senecio formosus* (“arnica”, “árnica de páramo”, “árnica de Bogotá”) is a plant from the Asteraceae (formerly known as Compositae) native to the highlands (3000–4000 meters above sea level) of Colombia, Venezuela, and Ecuador. In Colombia, it is commonly found in the Andean regions (moorlands) of the Departments of Cundinamarca, Cauca and Nariño where it can reach 60 to 120 cm in height. The plant grows in dense groups, it has channeled purple stalks, and its leaves are alternate, 8 cm in length by 2–3 cm wide. *S. formosus* has been traditionally considered as a medicinal plant in Colombia where its leaves are taken as an herbal tea, as a tincture or applied topically as a poultice [3,4]. Unfortunately, *S. formosus* leaves contain PAs and the intake of infusions made with dry leaves can cause severe veno-occlusive liver disease in humans [4].

In a single study, a total of 25 cases of hepatic veno-occlusive disease associated with the intake of *S. formosus* leaves were reported in Colombia [4]. All patients were jaundiced and ascitic and 13 of them died: 11 from liver failure, one from congestive heart failure and one from purulent meningoencephalitis [4]. Clinical laboratory studies showed moderate decrease in serum total protein content, slight elevation in serum liver amino transferases and increased serum bilirubin, particularly the conjugated fraction. In 80% of the patients, there was an increase in the serum activity of alkaline phosphatase.The main PA present in the dry leaves of *S. formosus* has been identified as retrorsine [4], a highly toxic retronecine-type cyclic diester PA capable of inducing hepatic CYP450 enzymes [19]. Lesser amounts of at least six more PAs (including integerremine) have also been found in dry leaves of *S. formosus* [4].

Fortunately, cases of human poisoning from *S. formosus* ingestion in Colombia have decreased since the 1997 report of Toro-González *et al.* [4]. From the 154 plant poisoning cases reported to the poison control center of the College of Medicine, National University of Colombia during the years 2006–2011, only three (1.9%) corresponded to *S. formosus* intake.

Another PA-containing plant that is sporadically associated with forensic cases in Colombia is *Symphytum officinale*, known with the common names of “consuelda”, “confrey” or “cofrey” [1,3]. *S. officinale* is traditionally used as a medicinal herb to treat wounds, inflammation, bruises, and thrombophlebitis [20]. The dry leaves are also used as a decoction to control mouth ulcers [20]. The wound healing effects of *S. officinale* have been demonstrated using an open wound model in rats [20]. Even though the plant is safe when used topically, it can produce irreversible hepatic damage if ingested chronically as an herbal tea [20]. There is almost no information about the composition of the *S. officinale* plants grown in Colombia, but a single Colombian sample analyzed for lycopsamine, echimidine and lasiocarpine showed only detectable levels of lycopsamine [21]; depending on the extraction method used, lycopsamine concentration ranged from 9.72 to 13.88 mg/kg (using pressurized hot water extraction) to 29.15 to 31.73 mg/kg (using hot reflux). Lycopsamine, a monoester PA, is one of the PAs most commonly detected in food [22]. Lycopsamine and related PAs occur in the Boraginaceae family and in the Eupatorieae (Asteraceae family) and its detection can be used as a marker for the undesirable presence of these plants in feed materials [22].

### 2.2. Pyrrolizidine Alkaloid Toxicosis in Domestic Animals

Toxicosis by PAs in domestic animals in Colombia has been reported in dairy cattle, goats, pigs, and poultry. The major plants involved in these cases have been *Senecio vulgaris*, *S. madagascariensis* and *Crotalaria* spp.

*Senecio vulgaris* (common groundsel) and *S. madagascariensis* (Madagascar ragwort, Madagascar grounsel) are tenacious annual or perennial weeds occurring in suitable disturbed habitats worldwide [5]. *S. madagascariensis*(“manzanilla del llano”) was reported for the first time in Colombia in the 1980s and has already colonized all the high plateau of the Departments of Cundinamarca and Boyacá [5]. *S. madagascariensis* plants collected in Colombia were found to contain chemical substances known as cacalolides [6]; however, the toxicological significance of these compounds remains to be determined. In another study conducted by our group, samples of *S. madagascarienses* collected in the Sabana de Bogotá area and analyzed for PAs in the Poisonous Plant Research Laboratory (Logan, UT, USA) showed a similar profile compared to plants from Australia or Hawaii although their PA concentration was lower [2]. The total concentration of PAs in samples from Australia, Hawaii and Colombia was 3089, 2133, and 805 μg/g, respectively. The major PAs found in the Colombian samples were senecivernine, senecionine, integerremine, mucronatinine, and usaramine. Interestingly, in dairy cattle farms located in the high plateau of the Departments of Cundinamarca and Boyacá, where both *S. madagascariensis* and *S. vulgaris* (“cerrajilla”, “yuyito”, “flor de piraguas”) are present, there have been reports of sudden death in cows immediately after parturition. The cause of this sudden death syndrome is unknown, but it is possible that the metabolic changes associated with parturition and the onset of lactation pose an extra load to a liver that has been severely affected by the chronic ingestion of the plants.

Another genus of PA-containing plants important in Colombia is the genus *Crotalaria* (Fabaceae), commonly known as rattlebox or crotalaria. In Colombia these plants are known with the common names of “crotalaria”, “cascabel”, “zapatico”, and “pajarito” [2]. They are herbs or shrubs that grow from 25 to 250 cm and can be grown as cover crops on sandy soils [2]. There are more than 600 *Crotalaria* spp. reported worldwide with at least 500 species reported in Africa. In Colombia, they grow from lowlands up to 3000 m above sea level particularly in areas with clearly defined dry periods, such as the inter-Andean valleys, the northern part of the country and the eastern savannas known as the llanos [2]. These plants may grow as weeds in well-fertilized soils used to cultivate soybean, sorghum and maize, and their seeds can contaminate these crops. At least 19 species of crotalarias are found in Colombia [11], including the widely recognized toxic species *C. spectabilis*, *C. retusa*, *C. sagittalis* and *C. pallida*.

Toxicosis due to *Crotalaria* spp. has been reported in goats, pigs, and chickens in Colombia. In 2001, a case associated with extremely high mortality rates in poultry and swine was reported in several farms located in the Department of Santander (northwest part of the country). The problem was associated with a sorghum cultivated in the northern part of the country that was used as an ingredient in swine and poultry mixed feeds. Samples of this sorghum were analyzed in the Toxicology Laboratory led by the author and were found to contain *Crotalaria* spp. seeds in a large percentage (2%–5%). Since the proper identification of the *Crotalaria* spp. cannot be done by seed morphology, the seeds were cultivated until flowering and were identified as *Crotalaria retusa* by botanists of the Colombian National Herbarium. The contamination levels found in the sorghum samples analyzed were extraordinarily high since it has been reported that a content of only 0.05% (equivalent to one *C. retusa* seed per 65,000 sorghum seeds) is associated with lethality in pigs [23]. *C. retusa* seeds are considered to be the most toxic after *C. spectabilis* seeds and they contain mainly the PA monocrotaline with traces of other PAs such as spectabiline [24]. To the knowledge of the author, this was the first case of massive toxicosis associated with *Crotalaria* spp. in Colombia.

*Crotalaria pallida* is another toxic *Crotalaria* species present in Colombia. *C. pallida* is native to Africa and usually grows in warm, open areas and in arid and semiarid regions [25]. *C. pallida* has been used as green manure because it has a high quantitative potential to supply nitrogen to the succeeding crop [26]. This use, however, can cause spontaneous growth within the new crop (corn, soybean, sorghum, *etc.*) which may lead to crotalaria seed contamination in crops used as ingredients in animal feed. A natural outbreak of *C. pallida* poisoning in goats was reported in the Department of Santander, Colombia [12].

Experimentally, *C. pallida* seeds collected in the eastern plains of Colombia were found to be highly toxic to broiler chickens [13] but less toxic to laying hens [14]. In chickens, the presence of 1%, 2% or 3% *C. pallida* seeds in their diet for 21 days caused mortality (2.1%, 6.2% and 16.7%, respectively), decreased body weight gain and feed efficiency, and increased serum activity of ALT and GGT [13]. Relative lung, heart and spleen weights were all significantly increased by 2% and 3% *C. pallida* seeds; the relative weight of the liver was significantly increased by 2% *C. pallida* seeds but significantly decreased by 3% dietary seeds [13]. Laying hens fed the same levels of *C. pallida* seeds in their diets for 35 days did not suffer mortality, but all dietary levels caused a significant decrease in body weight and feed intake. Egg mass production and average egg weight were significantly decreased by the supplementation of 2% and 3% *C. pallida* seeds. All levels of *C. pallida* significantly increased relative lung weight and 3% *C. pallida* seeds significantly decreased liver weight. *C. pallida* seed supplementation also increased significantly the activity of three of the four serum enzymes indicative of liver damage evaluated. Changes in the liver and lungs and ovarian regression were the major gross pathological findings in hens exposed to *C. pallida* seeds [14]. *C. pallida* seeds collected in Colombia were found to contain the PA usaramine and its corresponding *N*-oxide [14]. The concentration of usaramine was 0.16% and that of the *N*-oxide was 0.02% for a total PA content of about 0.18% (dry weight basis). When laying hens were fed 1%, 2% or 3% *C. pallida* seeds in their diets, usaramine was transferred into their eggs in a dose-dependent fashion [14]. The lowest level (1%) did not affect the layer’s performance but still resulted in usaramine levels in eggs above the maximum PA concentration recommended in some countries [14].

An interesting case of PA toxicosis in laying hens occurred in southern Colombia (Caloto, Cauca) in 2011 where 420,000 out of 2,200,000 (19.1%) layers were affected. High mortality was recorded (up to 19%) with decreased egg production, pale combs and depression. At postmortem examination, the birds showed lesions in the liver including increased size, increased friability and hemorrhaging. Two feed samples were analyzed for PAs and were found to contain 0.38 and 0.48 mg/kg of the PA seneciphylline. The major feed ingredients had been imported from Argentina and were soybean meal, full-fat soybean, yellow corn, sunflower meal, and wheat middlings. Seneciphylline has been reported in several *Senecio* species including *Senecio longilobus* [27] and *Senecio vulgaris* [28].

Another important genus containing PA is *Heliotropium*. There are between 250 and 300 species worldwide and the name of the genus derives from the fact that their leaves are constantly oriented towards the sun [2]. In Colombia, there are at least nine species of *Heliotropium*, all collectively known with the common name of “rabo de alacrán” (scorpion tail) due to the shape of the flowering stems [7]. Among these are two species that are well known for their toxic effects: *H. europaeum* and *H. indicum*. The main alkaloid in *H. indicum* is known as indicine *N*-oxide and has been shown to have low toxicity for rodents and other animal species [2]. In studies conducted in dogs and primates, it has been shown that indicine *N*-oxide causes reversible hepatic lesions (increase in serum levels of aspartate and alanine amino transferase and increased retention of bromosulfthalein) but no histologic lesions [8]. However, even though the international literature reports low toxicity of *H. indicum*, it is important to determine the toxicity of the plants that grow in Colombia, since in some regions of the country the plant has been related with photosensitization in cattle, an effect that could be attributed to PA toxicosis. In addition, it is important to establish whether animals exposed to this plant are exposed to other PA containing plants which may generate additive or synergistic interactions. In addition to PAs, *H. indicum* growing in Colombia has been shown to contain toxic levels of nitrate. Samples collected in the Departments of Córdoba and Sucre contained 178 and 7195 ppm of nitrate during the dry season and start of the rainy season, respectively [9]. Nitrate levels above 5000 ppm are considered toxic for cattle.

It is unknown whether other *Heliotropium* spp. present in Colombia (*H. angiospermum*, *H. peruvianum*, *H. procumbens*, *H. salicioides*, *H. ternatum*, *etc.*) accumulate PAs or if they present a toxicological risk for animals or humans.

## 3. Tropane Alkaloids

Several plant species capable of synthesizing tropane alkaloids are present in Colombia, including the closely related species of the Solanaceae family *Brugmansia* spp. and *Datura* spp., and the famous solanaceae *Atropa belladona*.

The genus *Brugmansia* includes seven species of large shrubs or small trees, with semi-woody and branched trunks [1]. They can grow between three and 11 meters tall and their leaves are alternate and often covered with fine hairs [1]. Their flowers are fragrant, pendulous (not erect) and their fruits have no spines [1]. The *Brugmansia* species reported in Colombia include *B. arborea*, *B. aurea*, *B. sanguinea*, *B. suaveolens*, *B. versicolor*, and *Brugmansia x candida*. All are present in the Andean highlands and adjacent areas (altitudes of 600–3700 m) and all receive the common name of “borrachero” [1]. Interestingly it has been reported in historic chronicles that the Chibcha Indians of the Colombian Andes (now all extinct) used to administer extracts of “borrachero” called “tonga” to the wives and slaves of a departed husband or master and then bury them alive with the deceased [29].

*Datura* species are herbaceous bushes with erect (not pendulous) flowers and most have spines on their fruits. *Datura* species reported in Colombia include *D. ferox*, *D. inoxia*, *D. metel* and *D. stramonium* [1]. *D. ferox* and *D. stramonium* (“chamico”) are mostly found in the Andes at altitudes of up to 1000 and 2600 m, respectively. *D. inoxia* and *D. metel* are found in the Caribbean plains (north of the country) and the Magdalena River valley at altitudes of 50–800 m and 0–200 m, respectively.

Both *Brugmansia* and *Datura* species accumulate tropane alkaloids, especially hyoscyamine (which after isolation forms a diastereomeric mix called atropine) and hyoscine, also known as scopolamine. Atropine is anticholinergic and causes blurred vision, salivation suppression, vasodilation, increased cardiac rate and delirium. Atropine is used in the treatment of toxicosis by parasympathetic chemicals like organophosphate and carbamate pesticides. Scopolamine is an antimuscarinic compound and smooth muscle relaxant. Tropane alkaloids are present in all parts of the plants and their concentration is greater in younger plants; however, the concentration of each alkaloid varies greatly depending on the specific type of plant. For example, atropine is more abundant in *D. stramonium* whereas scopolamine is prevalent in *D. ferox*. *D. stramonium* seeds found as contaminants in a soybean crop contained atropine, scopolamine and total alkaloid levels of 0.29%, 0.05%, and 0.34%, respectively, corresponding to an scopolamine:atropine ratio of 15:85 [30]. In another study, the scopolamine:atropine ratio found in *D. ferox* seeds was found to be 98:2 [31].

Humans can suffer from tropane alkaloid poisoning by accidental or deliberate exposure to *Brugmansia* seeds or flowers, whereas animals can be exposed from *Datura* spp. seeds contaminating crops such as soybean, corn or sorghum [2]. Toxicosis due to the intake of the fresh plant is very uncommon in horses and cattle due to the unpleasant odor and flavor of the plants [2]. Signs and symptoms of tropane alkaloid toxicosis include increased respiratory and cardiac rates, mydriasis, mouth dryness, thirst, diarrhea, confusion, hallucinations, ataxia, convulsions and, in severe cases, death from respiratory failure [2].

In Colombia, toxicosis in humans occurs due to exposure to extracts of *Brugmansia* spp. given by criminals to his victims [15]. These extracts are known commonly as “burundanga” and are rich in scopolamine but also contain atropine [15]. Scopolamine inhibits muscarinic receptors for acetylcholine and affects mainly neurotransmission pathways involved in memory. This effect causes memory loss of the specific event (lacunar amnesia), in addition to all the symptoms previously mentioned. Uribe *et al.* [15] reviewed the clinical histories of 860 patients admitted to a toxicology clinic in Bogotá, Colombia, after having been intoxicated with “burundanga”. Most of the patients were between 20 and 50 years old and the majority were males (79.1%); 67.4% of these patients were robbed.

## 4. Piperidine Alkaloids

Piperidine alkaloids contain a saturated heterocyclic ring (known as piperidine) in their structure. The best known piperidine alkaloids are those from *Conium maculatum* L. (poison hemlock) [2]. This plant from the Apiaceae family is fairly well known as it was used as the poison of the state in ancient Greece. In Colombia, parts of the plant have been found in post-mortem examination of suicide cases in Colombia, and it has also been associated with deaths due to the intake of plant leaves as an herbal tea [3]. *C. maculatum* is known in Colombia as “cicuta”, “perla” or “angélica” and currently is a naturalized plant, very common in the high plains of the Sabana de Bogotá where it is sometimes used as ornamental. *C. maculatum* contains at least five piperidine alkaloids, the most toxic being coniine (especially in the seeds) and γ-coniceine (in vegetative tissues). The other three alkaloids are *N*-methyl-coniine, conhydrine and pseudoconhydrine. Coniine was the first alkaloid to be chemically synthesized in 1886. Signs and symptoms of *C. maculatum* toxicosis in animals and humans have been reviewed by Panter *et al.* [32] and Vetter [33]. Coniine, γ-coniceine and *N*-methylconiine cause paralysis of the musculature due to the blockade of the neuromuscular junctions [2]. The initial signs of the acute toxicosis include muscle weakness, tremors, incoordination and mydriasis, followed by bradycardia, depression, coma and death from respiratory failure [2]. Conium alkaloids can also cause congenital malformations in calves including cleft palate and musculoskeletal contractures. Poultry species (turkeys, geese, and quail) show ataxia and inability to fly [34]. The closely related toxic plant of the same family (Apiaceae), known as water hemlock (*Cicuta*s pp.), has not been reported in Colombia.

## 5. Indolizidine Alkaloids: Swainsonine

Swainsonine is a neurotoxic indolizidine alkaloid first isolated from plants of the *Swainsona* genus, hence the name [2]. Swainsonine inhibits lysosomal hydroxylases, especially α-mannosidase, causing a cellular alteration known as lysosomal storage disease and characterized by excessive carbohydrate accumulation within lysosomes [35]. *Ipomoea carnea* (batatilla) is common in the Colombian eastern plains, and it is one of the plants known worldwide to be capable of accumulating swainsonine, along with plants of the genera *Astragalus*, *Oxytropis*, *Sida*, and *Swainsona* [2]. *I. carnea* is native to tropical and subtropical America and grows spontaneously as a weed in pastures in the eastern plains and other parts of the country; it is also used as ornamental for its colorful flowers. When present as a weed in pastures, it may cause adverse effects in cattle, horses and goats including decreased weight and neurological alterations including failure to take and swallow feed, hypermetria and ataxia [36,37]. No postmortem changes are observed but histological lesions in neurons are reported. *I. carnea* is regarded as one of the most important toxic plants for cattle raised in the Arauca River valley of Colombia [10]. It has been shown that swainsonine in *Astragalus* and *Oxytropys* species is produced by an endophyte fungus, and it is possible that a swainsonine-producing endophyte may be associated with *I. carnea* as well [38]. If this is true, reducing the endophyte in the plant will also reduce its toxicity.

## 6. Final Comments

Given the fact that Colombia has the second largest plant biodiversity in the world and that many plant families contain potentially toxic alkaloids, it is not surprising that toxicosis due to toxic plants containing alkaloids is commonly reported in Colombia. Unfortunately, few cases are documented in the scientific literature. Toxic plant research in Colombia is only in its infancy and there are only a few research groups working on toxic plants in the country. More research is needed with the support of government funding in order to develop the instrumental infraestructure for the determination of toxins in plants and, for the creation of interdisciplinary research groups involving veterinarians, botanists, analytical chemists, and other professionals interested in this important field of research.
